# Cracked titanium film on an elastomeric substrate for highly flexible, transparent, and low-power strain sensors

**DOI:** 10.1186/1556-276X-8-441

**Published:** 2013-10-24

**Authors:** Jin-Seo Noh

**Affiliations:** 1 Department of Nano-Physics, Gachon University, 1342 Seongnamdaero, Seongnam-si, Gyeonggi-do 461-701, South Korea

**Keywords:** Titanium film, Polydimethylsiloxane, Cracks, Strain sensor

## Abstract

Strain-dependent cracking behaviors in thin titanium (Ti) films on polydimethylsiloxane (PDMS) substrates were systematically investigated for their application to sensitive, flexible, transparent, and portable strain sensors. When uniaxially elongated, vertical cracks were developed in the low-strain range, and beyond a critical strain, tilted cracks appeared to intersect the vertical cracks. The cracking behaviors were also dependent on Ti film thickness. The varying strain-dependent crack patterns produced a significant resistance change in response to the applied strain, particularly, in the high- and broad-strain range. For a 180-nm-thick Ti film on PDMS substrate, a gauge factor of 2 was achieved in the range of 30% to 50% strain. The operation power was extremely low. All the Ti films on PDMS substrates were transparent, highly flexible, and very easy to fabricate. These results suggest that cracked Ti films on PDMS substrates could be a viable candidate for realizing a low-cost, flexible, transparent, and portable strain sensor.

## Background

Measuring strain accurately has become much more important since new technology fields such as health monitoring, artificial skin engineering, intelligent textile engineering, motion detection, and environment monitoring have emerged [1-7]. Flexible materials are widely employed for these applications due to the diversity of body shapes to which the sensors are attached and the variability of strain in action. Recent progress on the material systems includes graphene ripples on polydimethylsiloxane (PDMS) substrates [8], Si/Ge nanowire matrix on polyimide substrates [3], Pt-coated polymer nanofibers sandwiched between PDMS sheets [9], Si nanoribbons on polyimide substrates [10], carbon nanotube ribbons embedded in PDMS [11], ZnO nanowire/polystyrene hybrid structure on PDMS [12], and graphene on PDMS [13]. Although high gauge factors reaching 116 and the adaptability to various forms of stresses such as tension, compression, shear stress, and torsion have been demonstrated through those approaches, a few weak points still need to be addressed. For instance, sensor fabrication processes were somewhat complicated, tolerable strains were low (less than several percent) for many systems, and most sensors were not completely transparent, whereas conventional strain sensors made of metal foils also suffer from limited sensitivity and high power consumption [14].

From previous works on palladium (Pd) film on a PDMS substrate, it was demonstrated that the Pd film was broken into pieces under an external or internal strain and it could be applied for highly sensitive hydrogen gas sensors [15-18]. A mechanism by which nanocracks are generated in the Pd film on PDMS substrate was proposed, and a general process on how the nanocracks thus generated respond to hydrogen molecules was also provided [15,17]. However, systematic studies on the expandability of the proposed mechanism to other metals and the crack generation behaviors dependent on the magnitude of applied strain were missing.

In this work, we investigated the effect of applied strain and film thickness on nanocrack generation using titanium (Ti) films on PDMS substrates. Ti was chosen as the film material because of its several advantages such as good adhesion to diverse materials, high strength-to-weight ratio, good resistance to corrosion, and high biocompatibility even though it is a poor conductor [19-22]. Differing patterns of cracks in the Ti film created under varying strains resulted in a change in electrical resistance that corresponded to the applied strain, providing an opportunity that the cracked Ti film on PDMS substrate could be used for a flexible strain sensor covering a wide range of strain. The suggested strain sensor is very easy to fabricate and handle, which ultimately allows for low-cost, portable strain sensors. It is also transparent, thereby expanding its potential use to monitoring deformations in various transparent bodies such as fragile structures, flexible electronics, and health-monitoring appliances.

## Methods

**Figure 1:**
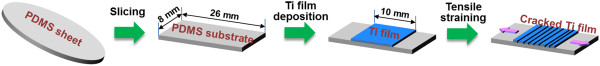
**Schematic process to fabricate a cracked Ti film on a PDMS substrate.** Step 1: preparation of a PDMS sheet, step 2: slicing of the PDMS sheet into 26 mm × 8 mm-sized samples, step 3: deposition of a Ti thin film on the PDMS substrate, and step 4: generation of cracks by mechanical stretching.

A schematic procedure to fabricate a cracked Ti film on a PDMS substrate is illustrated in Figure 1. To prepare an elastomeric PDMS sheet, a PDMS base resin (Sylgard 184, Dow Corning, Midland, MI, USA) was first mixed with a curing agent (Dow Corning) in a vial at a fixed weight ratio (10:1), and the mixture was poured onto a petri dish followed by degassing for more than 1 h [16,23]. It was then cured at 70°C for 3 h [16], and the sheet thickness was 0.4 mm after curing. The cured PDMS sheet was sliced into a size of 28 mm (length) × 8 mm (width) rectangular samples. Ti films were deposited on the PDMS substrates by radio-frequency (RF) sputtering using a 2-in. Ti target (purity 99.99%). The base pressure was kept below 10^-6^ Torr. Film deposition was performed in an Ar gas flow of 9 sccm (process pressure approximately 1 × 10^-3^ Torr) at a RF power of 50 W. In this condition, the film growth rate was approximately 4 nm/s, and Ti films of varying thicknesses (80, 180, and 250 nm) were grown on the PDMS substrates with controlled deposition time. The Ti film area was constrained to 10 mm (length) × 8 mm (width) by masking both ends of the PDMS substrates during deposition. In the next step, the Ti films on PDMS substrates were uniaxially elongated to induce cracks in the Ti films. Here, the magnitude of applied strain was modulated in the range of 0% to 80%.

The strain-dependent cracking behaviors of the Ti films on PDMS substrates were examined first at the microscale using an optical microscope (Olympus BX 51,Olympus Corporation, Tokyo, Japan). To stereoscopically investigate the patterns and sizes of the cracks at the smaller scale, the samples were three-dimensional (3D)-scanned using a 3D laser scanning microscope (Olympus CLS 4000). In addition, scanning electron microscopy (SEM, Hitachi S4800, Hitachi High-Tech, Tokyo, Japan) was utilized to closely observe individual cracks. The resistances of the cracked Ti films on PDMS substrates were measured by a simple two-probe method, using a probe station connected to a high-resolution, multi-purpose electrical characterization system (Keithley 4200-SCS, Keithley Instruments Inc., Cleveland, OH, USA). The extremely high-resolution system enabled to detect a femto-ampere-level current and to measure a resistance of more than 1 TΩ. The resistance was monitored not only under normal tension, but it also measured under non-planar straining along a curved surface.

## Results and discussion

**Figure 2:**
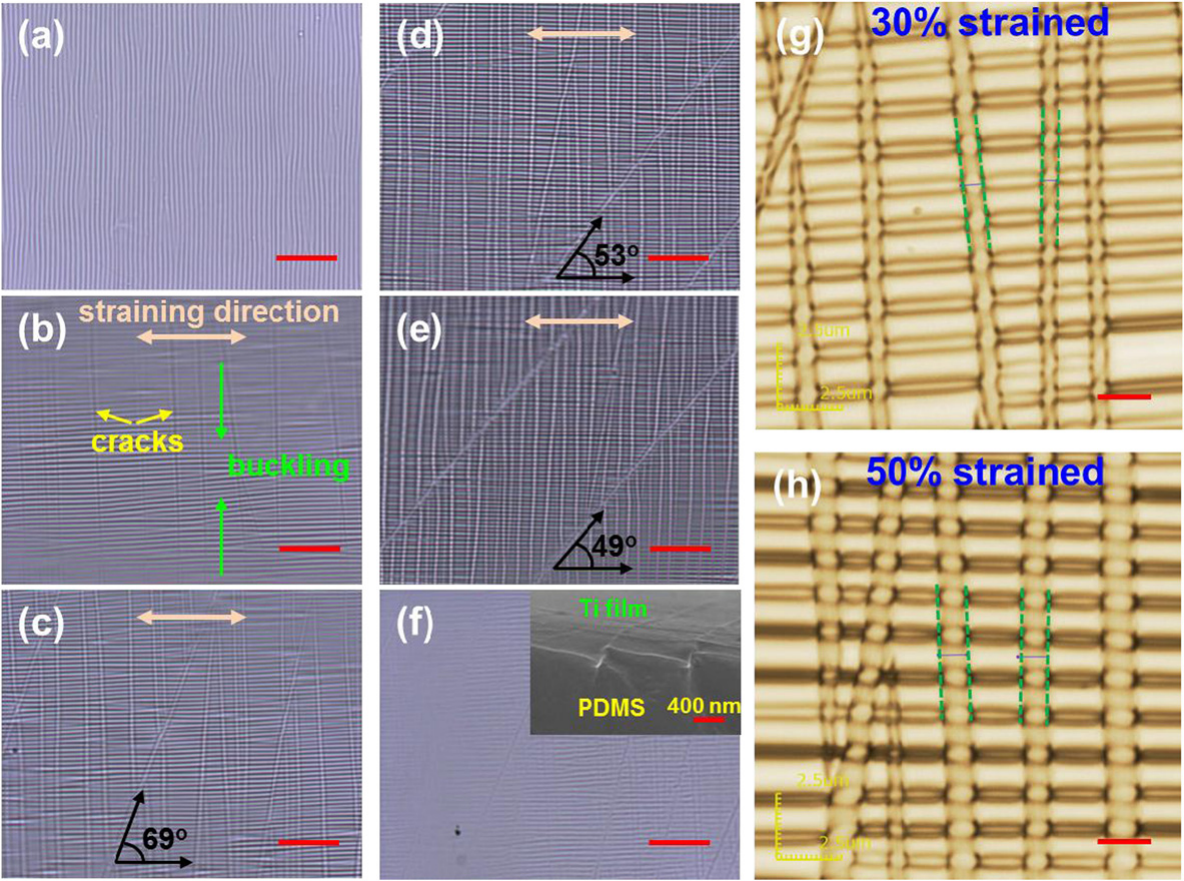
**Optical microscope images of a 180-nm-thick Ti film on PDMS substrate. (a)** Before straining, under different uniaxial strains of **(b)** 10%, **(c)** 30%, **(d)** 50%, **(e)** 80%, and **(f)** after strain relaxation. The inset in (f) is a SEM image of the sample after strain relaxation. In (b), the straining direction and the presence of both vertical cracks and buckling are indicated, and in (c, d, e), the straining direction and angles between the secondary cracks and the straining direction are shown. LSM images of the sample at **(g)** 30% and **(h)** 50% strain. Green dotted lines are shown to estimate the average crack widths at the respective strains. Scale bars are 20 μm for **(a**, **b**, **c**, **d**, **e**, **f)** and 2 μm for **(g)** and **(h)**.

Figure 2a,b,c,d,e,f shows optical microscope images of a 180-nm-thick Pd film on the PDMS substrate, which were obtained under a tensile strain of 0% (Figure 2a), 10% (Figure 2b), 30% (Figure 2c), 50% (Figure 2d), 80% (Figure 2e), and after strain relaxation (Figure 2f). Here, the strain is a length change normalized to the original length, which is simply expressed as *ϵ* = (*L*- *L*_0_)/*L*_0_ × 100%, with *L*_0_ and *L* being the original length and the length under a strain, respectively. It is found from Figure 2a that fine ripples exist on the surface of the Ti film, presumably coming from the small residual strain of the PDMS substrate underneath. Upon applying a 10% strain, cracks begin to form in the direction perpendicular to the straining direction while buckling occurs at the same time due to the compressive stress acting perpendicularly to the direction of the tensile stress, as shown in Figure 2b. Based on the previous research, the cracks are initiated from the surface of PDMS substrate because the originally soft PDMS surface is modified to a silica-like hard surface during metal sputtering [15]. Once the cracks are initiated at the Ti/PDMS interface, they are supposed to propagate through the Ti film, but the most applied stress is likely to be consumed for PDMS surface cracking at low-strain levels. This is why the crack patterns are not very clear at 10% strain. The cracks become clearer as the strain level increases. This is confirmed by the images shown in Figure 2c,d,e. Interestingly, the secondary crack patterns that are tilted by certain angles from the vertically formed first cracks begin to appear from a 30% strain. The tilting angle becomes larger with increasing strain (21° to 41° in the strain range of 30% to 80%), reaching an angle of 49° between the crack lines and the straining direction at an 80% strain (Figure 2e). In an ideal case, under a uniaxial tension of *σ*_*x*_, the maximum shear stress, *τ*_max_ = *σ*_*x*_/2, acts at an angle of 45° with the stress direction [24]. It is speculated that the applied stress is dominantly exhausted to generate vertical cracks until reaching a critical stress, *σ*_*c*_ (or critical strain, *ϵ*_*c*_), and beyond *σ*_*c*_, the shear stress gradually plays a significant role, producing secondary cracks that deviate more and more from the first cracks with an increase in stress. The elongated film with cracks are mostly recovered to its original dimension after the strain is released, but indistinct crack lines are left as seen in Figure 2f. The inset of Figure 2f reveals that the cracks are closed after strain relaxation. The strain-dependent crack patterns were similarly reproduced even in the second strain cycle (not shown). For the second strain cycle, the tilting angle of the secondary cracks with respect to the vertical primary cracks showed a range of 19° to 40° for the applied strains of 30% to 80%, which is very close to that observed in the first strain cycle.

Although optical microscopy revealed the overall cracking behaviors of the Ti film on PDMS substrate, its resolution is limited and the data is two-dimensional. To overcome these shortcomings, laser scanning microscopy (LSM) was utilized. LSM images for a 180-nm-thick Ti film subjected to 30% and 50% strains, respectively, are presented in Figure 2g,h. Now, both cracks and buckling are seen much more clearly, and inter-crack distances are found to range from 1 to 4 μm, which are shorter than the average value estimated from the optical images. Comparing crack patterns created by the respective strains, the average crack width (1.09 μm) at 50% strain is larger than that (0.72 μm) at 30% strain, and the buckling density is also larger at a higher strain state. The inter-crack spacings are similar for both strain states.

**Figure 3:**
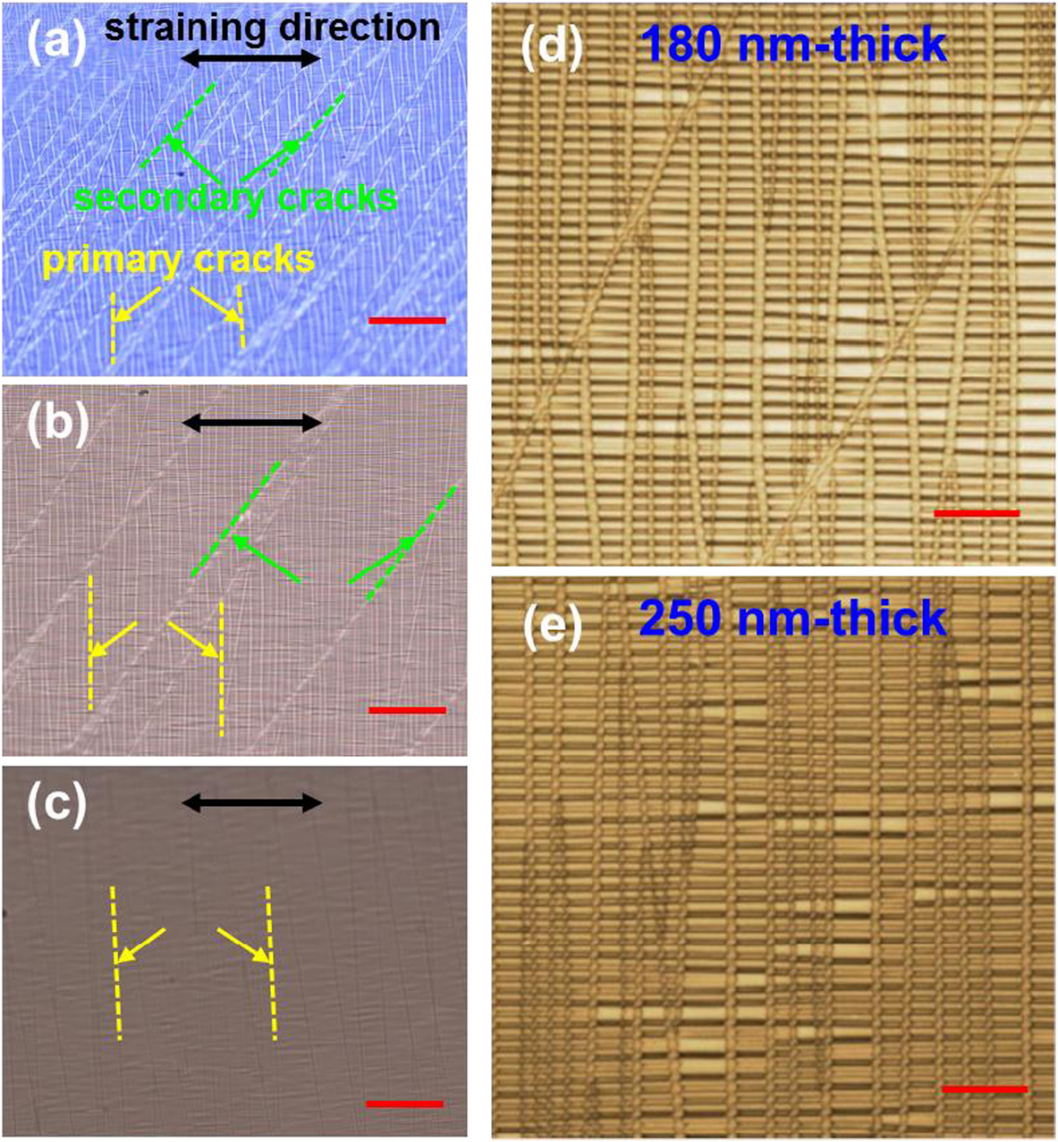
**Optical microscope and LSM images of Ti films on PDMS substrates at a strain of 50%.** Optical microscope images of **(a)** 80 nm, **(b)** 180 nm, and **(c)** 250 nm on PDMS substrates at an identical strain of 50%. In **(a**, **b**, **c)**, the straining direction and the directions of primary cracks and secondary cracks are displayed. LSM images of **(d)** 180-nm and **(e)** 250-nm Ti films on PDMS substrates at the same strain (50%). Cracks in the 250-nm sample look narrower compared to the 180-nm sample. Scale bars are 50 μm for **(a**, **b**, **c)** and 10 μm for **(d**, **e)**.

The Ti film thickness dependence of cracking behaviors was also investigated. Figure 3a,b,c shows optical micrographs of Ti films with thicknesses of 80 nm (Figure 3a), 180 nm (Figure 3b), and 250 nm (Figure 3c) on PDMS substrates under an identical strain of 50%. In Figure 3a, it is found that the relatively narrow-spaced, approximately 45°-tilted secondary cracks (represented by green dotted lines) intersect the vertically aligned primary cracks (represented by yellow dotted lines) that are formed beforehand. This observation is still effective in a 180-nm-thick Ti film, but the average distance between adjacent secondary cracks is much larger than in the 80-nm-thick Ti film (Figure 3b). The secondary cracks finally disappear when the Ti film attains a 250-nm thickness (Figure 3c). The absence of secondary cracks is further supported by the LSM images (see Figure 3d,e). In actuality, the average crack width in the 250-nm Ti film was measured to be 0.88 μm, which corresponds to a 20% reduction from the 180-nm Ti film. These are because more stress is expended in propagating cracks through Ti film for full development of the vertical cracks; thus the *σ*_*c*_ becomes larger as the film thickness increases. In this respect, the film thickness dependence of cracking is qualitatively consistent with the strain-dependent cracking explained above.

**Figure 4:**
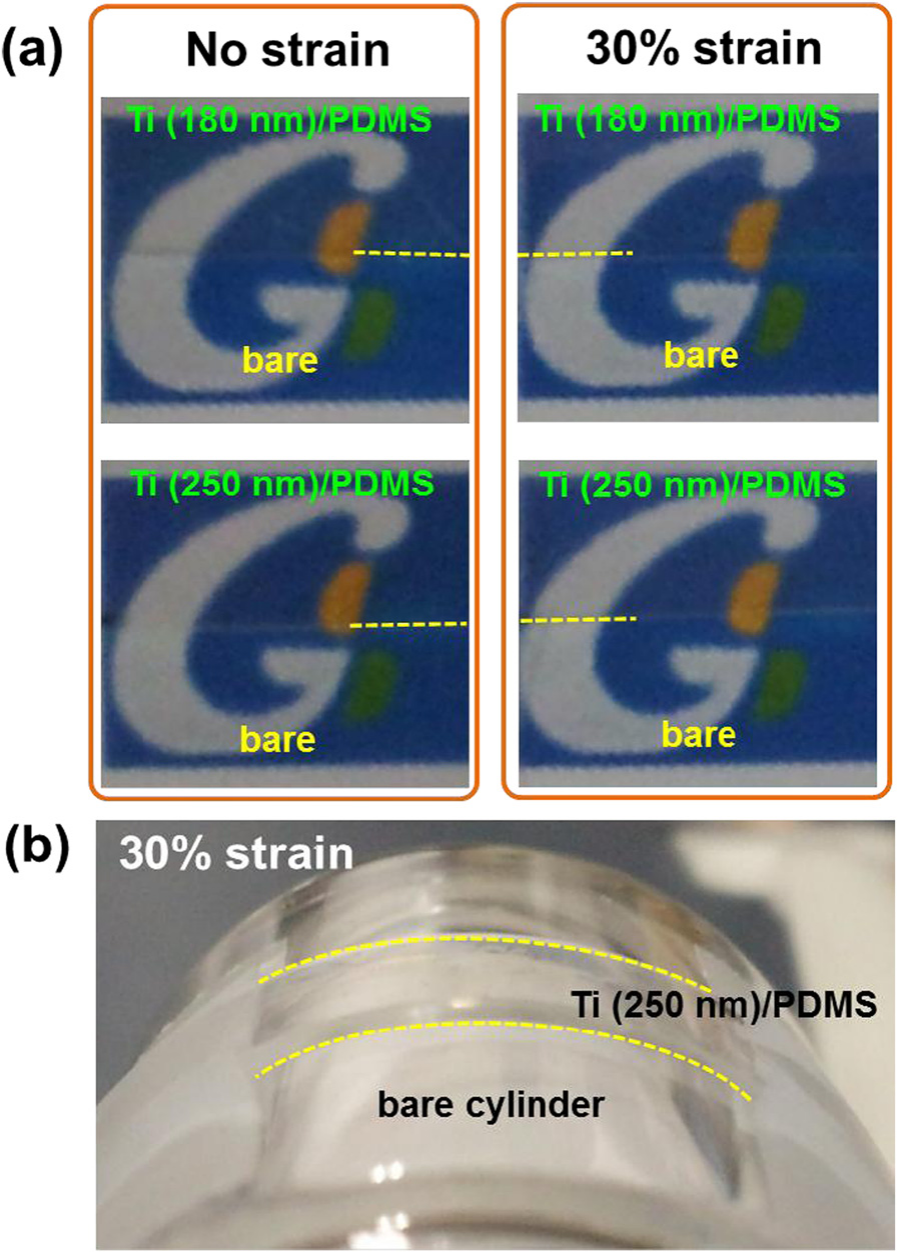
**Photographs showing the transparency of Ti films on PDMS substrates. (a)** Ti films with thicknesses of 180 nm (upper) and 250 nm (lower) on PDMS substrates at zero strain (left) and 30% strain (right) covering only half of the paper design underneath. **(b)** A 250-nm-thick Ti film on PDMS substrate wrapped around a transparent cylinder with a radius of curvature of 11 mm. Yellow dotted lines are drawn along the boundaries between the sample-overlaid areas and the bare areas.

All Ti films on PDMS substrates were transparent in the measured Ti film thickness range of 80 to 250 nm. Figure 4a shows the transparency of flat 180-and 250-nm-thick Ti films on PDMS substrates at both zero strain and 30% strain. Furthermore, the Ti films on PDMS substrates retained the transparency under the mixed stress state of bending and stretching, as shown in Figure 4b where a 250-nm-thick Ti film/PDMS sample was strained by 30% along the surface of a transparent cylinder with a radius of curvature of 11 mm. From these results, it is confirmed that Ti films on PDMS substrates are transparent irrespective of the strain state. The transparency of the Ti films on PDMS substrates offers a potential that they could be particularly considered for special applications such as flexible electronics, health monitoring, and transparent structure diagnostics.

**Figure 5:**
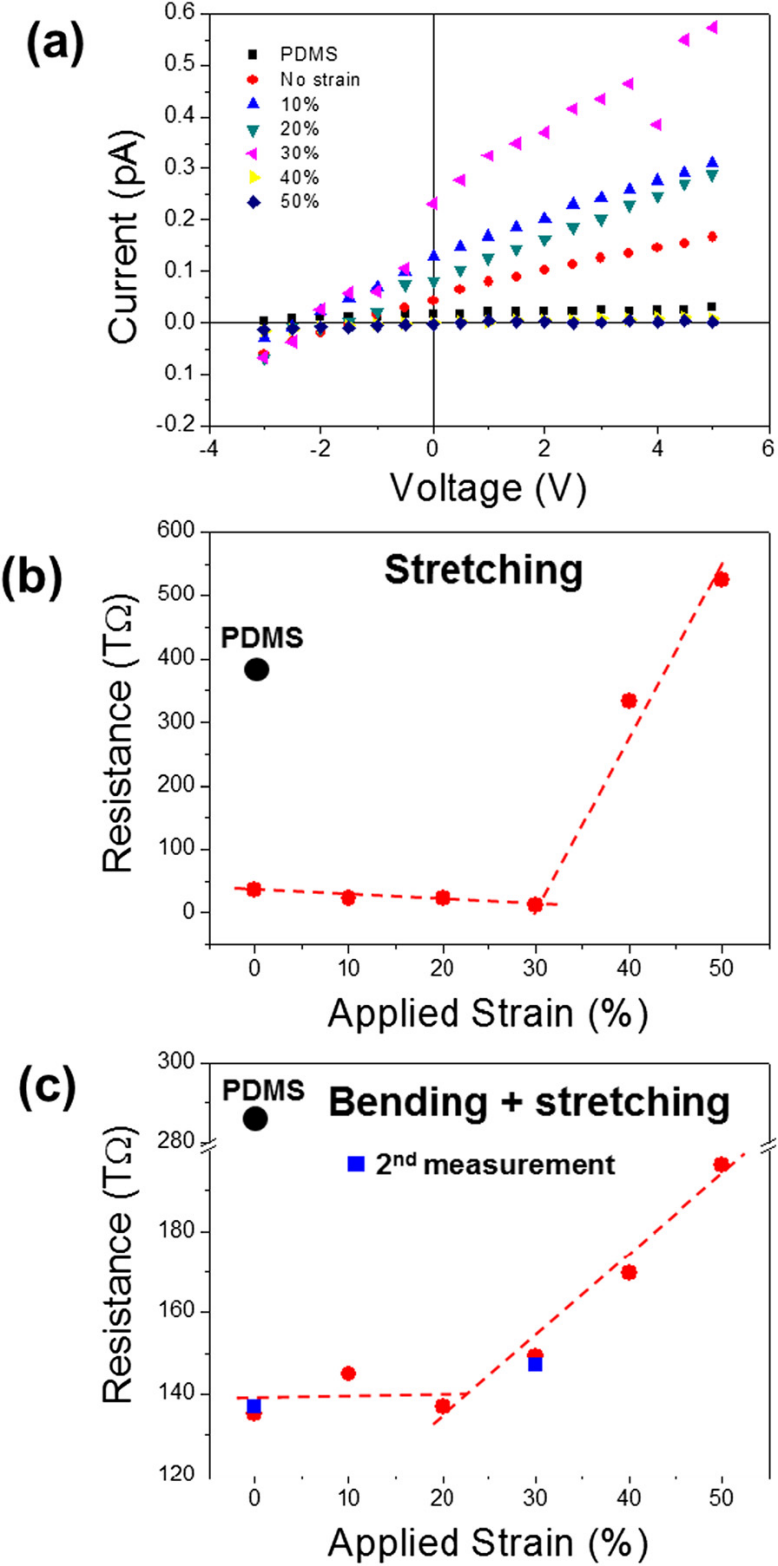
**Strain-dependent *I***-***V *curves and resistance versus strain plots. (a)** Strain-dependent *I*-*V* curves of a 180-nm Ti film on PDMS substrate. Here, the strain was applied by uniaxial stretching. *I*-*V* curve of a pure PDMS sheet is also shown for comparison. Resistance versus strain plots of the sample under **(b)** simple stretching and **(c)** mixed straining of bending and stretching. In **(c)**, blue square symbols represent resistances measured from the second straining cycle.

The resistances of the Ti films on PDMS substrates subjected to varying strains were measured by a simple two-probe method, using an ultrasensitive electrical characterization system. Figure 5a exhibits strain-dependent current (*I*) to voltage (*V*) curves obtained from a 180-nm-thick Ti film on the PDMS substrate. Here, the sample was uniaxially stretched. The curves are, in general, linear for all the measured strains (0% to 50%) although there appear slight offsets at the origin. The extremely small currents of less than 1 pA (= 1 × 10^12^ A) were thought to originate from a combination of the thin Ti film thickness and the possible surface oxidation of the Ti film into TiO_2_. From the slopes of the *I*-*V* curves, electrical resistances of the samples under different strains were calculated, and representative data for the uniaxially stretched 180-nm Ti/PDMS sample are presented in Figure 5b. The resistance of the unstrained Ti film on PDMS sample is approximately an order of magnitude smaller than that of a PDMS substrate. Upon application of a strain, the resistance changes. However, the resistance-changing trend is found to be not monotonic but divided into two regions: an almost steady region and a sharp-changing region. In the low-strain region, the resistance changes very little even under a significant amount of strain, while it rapidly increases with the increasing strain level in the high-strain region. In the high-strain region, the change in resistance per unit strain change, ∆*R*/∆*ϵ*, reaches 25.7 TΩ/% (= 2.57 × 10^13^ Ω/%). This resistance sensitivity to strain makes the cracked Ti film on PDMS substrate applicable to a strain sensor that can operate in the high- and broad-strain range. In this case, the sample gives the normalized resistance change to the unit strain change (so-called gauge factor), ∆*R*/(*R*_0_*·*∆*ϵ*) = 2.0, which is comparable to the values of conventionally used metals such as Cu, constantan, and Ag [10,25,26]. In contrast to the conventional strain-sensing materials of which ultimate strain is limited to <1%, the cracked Ti film on the elastomeric substrate shows much higher strain tolerances up to 50% and a broader sensing range of 30 to 50%. In addition, the power consumption of the sample is extremely small (<3 pW) in the measured range, which is a great advantage for portable strain sensors.

The cracked Ti film on PDMS substrate can also endure a mixed stress state since it is very flexible. Figure 5c shows a resistance versus strain plot obtained from the 180-nm Ti film on PDMS substrate wrapped around a cylinder with a radius of curvature of 11 mm (see Figure 4b). In this case, strains were applied along the curved surface, forcing a combination of bending and stretching to the sample. Like the simple stretching case, the resistance-changing trend is divided into a steady region (low-strain region) and a sharp-changing region (high-strain region). In the high-strain region, the ∆*R*/∆*ϵ* is approximately 2.0 TΩ/%, which is far smaller than under simple stretching. When measured again after relaxation of the applied strain, the resistance at each strain was reproducible as shown by the blue square symbols in Figure 5c. It is not clear at this moment why the resistance-changing trends are divided into two regions for both simple stretching and more complex straining of bending and stretching. A clue, however, can be deduced from the cracking behavior of the sample. The border between the two regions exists around a 30% strain for the 180-nm-thick Ti/PDMS sample, coinciding with the initiation point of the tilted secondary cracks (*ϵ*_*c*_ ≈ 30%). It is inferred that below this strain, the vertical cracks are not fully developed and there is still a connected current path, and then all the current paths are severed with the advent of the secondary cracks above the critical strain, which causes a steep resistance increase with a small increase in strain. This was supported by the fact that no significant resistance variation was observed in the strain range of 0% to 50% for a 250-nm-thick Ti film on PDMS substrate, where only weak vertical cracks appear. Despite many advantages of the cracked Ti film on PDMS substrate as a strain sensor, there still remain issues to be further addressed, including the effects of irregular crack patterns and surface oxide and how to widen the strain-sensing range more, particularly toward the lower strains.

## Conclusions

Thin Ti films with thicknesses of 80 to 250 nm were sputter-deposited on elastomeric PDMS substrates. All the samples were transparent and highly flexible. Cracks were introduced in the Ti films by both planar and non-planar stretching, but the cracking behaviors differed depending on the applied strain and the Ti film thickness. Vertical cracks were developed at low strains below a critical strain, and beyond it, secondary cracks tilted from the straining direction appeared to intersect the earlier formed vertical cracks. The strain-dependent crack patterns led to the strain-dependent resistance. For a 180-nm Ti film on PDMS substrate, a sharp-resistance-changing region appeared over a tensile strain range of 20% above a critical strain of 30%, where a gauge factor of 2 was achieved. It also showed extremely low-power consumption and endured a mixed strain of bending and stretching. These attributes of cracked Ti films on PDMS substrates provide a pathway for the embodiment of an advanced strain sensor with low-cost manufacturability, high transparency and flexibility, and good portability.

## Competing interests

The author declares that he has no competing interests.

## Author’s information

JSN earned his Ph.D. degree in materials science in 2003 from University of Wisconsin-Madison. He has been with the Samsung Advanced Institute of Technology (SAIT) as a member of the research staff from 2003 to 2008 and with the Institute of Nanoscience and Nanotechnology of Yonsei University as a research professor from 2009 to 2012. Now, he is an assistant professor in the Department of Nano-physics of Gachon University. His research interests include nanomaterial-based thermoelectric energy conversion, nanostructure-utilizing gas sensors and physical sensors, nanoelectronics/spintronics, and technology fusion crossing the borders.
